# High-resolution metabolomics to discover potential parasite-specific biomarkers in a *Plasmodium falciparum* erythrocytic stage culture system

**DOI:** 10.1186/s12936-015-0651-1

**Published:** 2015-03-24

**Authors:** Youngja H Park, Ya Ping Shi, Bill Liang, Carl Angelo D Medriano, Young Ho Jeon, Eucaris Torres, Karan Uppal, Laurence Slutsker, Dean P Jones

**Affiliations:** Dept of Medicine, Emory University School of Medicine, Atlanta, GA 30322 USA; College of Pharmacy, Korea University, Sejong City, South Korea; Malaria Branch, Division of Parasitic Diseases and Malaria (DPDM), Centers for Disease Control and Prevention (CDC), Atlanta, USA

**Keywords:** Malaria, *Plasmodium falciparum*, Mass spectrometry, High-resolution metabolomics (HRM), Biomarkers, Diagnostic tool

## Abstract

**Background:**

Current available malaria diagnostic methods each have some limitations to meet the need for real-time and large-scale screening of asymptomatic and low density malaria infection at community level. It was proposed that malaria parasite-specific low molecular-weight metabolites could be used as biomarkers for the development of a malaria diagnostic tool aimed to address this diagnostic challenge. In this study, high resolution metabolomics (HRM) was employed to identify malaria parasite-specific metabolites in *Plasmodium falciparum in vitro* culture samples.

**Methods:**

Supernatants were collected at 12 hours interval from 3% haematocrit *in vitro* 48-hour time-course asynchronized culture system of *P. falciparum*. Liquid chromatography coupled with high resolution mass spectrometry was applied to discover potential parasite-specific metabolites in the cell culture supernatant. A metabolome-wide association study was performed to extract metabolites using Manhattan plot with false discovery rate (FDR) and hierarchical cluster analysis. The significant metabolites based on FDR cutoff were annotated using Metlin database. Standard curves were created using corresponding chemical compounds to accurately quantify potential *Plasmodium*-specific metabolites in culture supernatants.

**Results:**

The number of significant metabolite features was 1025 in the supernatant of the *Plasmodium* infected culture based on Manhattan plot with FDR q=0.05. A two way hierarchical cluster analysis showed a clear segregation of the metabolic profile of parasite infected supernatant from non-infected supernatant at four time points during the 48 hour culture. Among the 1025 annotated metabolites, the intensities of four molecules were significantly increased with culture time suggesting a positive association between the quantity of these molecules and level of parasitaemia: i) 3-methylindole, a mosquito attractant, ii) succinylacetone, a haem biosynthesis inhibitor, iii) S-methyl-L-thiocitrulline, a nitric oxide synthase inhibitor, and iv) O-arachidonoyl glycidol, a fatty acid amide hydrolase inhibitor, The highest concentrations of 3-methylindole and succinylacetone were 178 ± 18.7 pmoles at 36 hours and 157±30.5 pmoles at 48 hours respectively in parasite infected supernatant.

**Conclusion:**

HRM with bioinformatics identified four potential parasite-specific metabolite biomarkers using *in vitro* culture supernatants. Further study in malaria infected human is needed to determine presence of the molecules and its relationship with parasite densities.

**Electronic supplementary material:**

The online version of this article (doi:10.1186/s12936-015-0651-1) contains supplementary material, which is available to authorized users.

## Background

Among four species of human malaria parasites, *Plasmodium falciparum* is responsible for most malaria-attributed morbidity and mortality. Over the past decade, successful scale-up of malaria control has resulted in substantial reductions in malaria cases and deaths [1,2]. As malaria transmission decreases due to control efforts, the epidemiology of malaria may change; that is, an increasing proportion of infections at the community level may be asymptomatic and of low parasite density [3,4]. Current malaria diagnostic tools include: 1) parasite detection by microscopic examination of blood smears, 2) antigen-based rapid diagnostic tests (RDTs), and 3) sensitive DNA-based assays. All these diagnostic methods require blood sampling by finger-prick and their implementation has been limited by either their labour/time intensive nature and requirement for specialized training and skills (microscopic method), moderate sensitivity (RDTs, microscopy), or high cost of sample preparation and supporting infrastructure needed (DNA-based methods). For programmes aiming to reduce transmission by further decreasing the parasite reservoir in humans through large scale screening approaches to detect and then to radically cure asymptomatic low-density malaria infections in real time, a field-deployable non-invasive, sensitive, low-cost, simple diagnostic tool would be very useful at the community level. Currently, available diagnostic tools cannot meet this challenge. Therefore, it was proposed to identify malaria parasite-specific low molecular-weight metabolites that could potentially be used for future development of such diagnostic tools.

As the first step for proof of concept, this study was designed to identify parasite specific low molecular-weight metabolites in an *in vitro* 48-hour time-course culture system of *P. falciparum* using high-resolution metabolomics (HRM) [5-7]. Earlier metabolomic studies on malaria have mainly focused on metabolic pathways and enzymes for the development of therapeutic strategies and interpretation of malaria pathogenesis. For example, using metabolomics with LC-MS/MS, Olszewski *et al*. identified the potential mechanism of cerebral malaria pathogenesis which was associated with the depletion of arginine [8]. More recently, NMR techniques in malaria metabolite profiling has been applied to identify biomarkers for infections. Tritten *et al*. identified two urinary metabolites in *Plasmodium berghei* infected mice, while a study by Sengupta et al. suggested urinary ornithine seems to have the potential as biomarkers of *Plasmodium vivax* malaria [9,10]. Although the two studies were conducted in rodent malaria and *P. vivax* malaria respectively, their analytical approaches could be used in mining for biomarkers of *P. falciparum* malaria infection. A study by Teng *et al.* showed strain-specific differences in a range of metabolites in erythrocytes infected with *P. falciparum* from *in vitro* culture and further highlighted the variation in levels of choline and phosphocholine among the strains [11]. In addition, Sana *et al*. investigated global mass spectrometry-based metabolomic profiling between *in vitro P. falciparum* infected and uninfected erythrocytes [12]. They demonstrated the alteration of metabolic profiling including the glycolysis pathway and tricarboxylic acid (TCA) cycle elucidating the mechanism of host-parasite interactions.

In contrast to the above reports, this study was designed to explore *P. falciparum* specific waste products, low molecular-weight metabolites, in the supernatant from the erythrocyte culture system. The rationale for profiling low molecular-weight metabolites in culture supernatants, first, was based on the hypothesis that parasite-specific small molecular wastes could be secreted into urine, saliva or sweat at high concentrations in malaria infected human and the ultimate goal is to use the small molecules as potential biomarkers for development of non-invasive and sensitive malaria diagnostic tools. This study demonstrated HRM could achieve a relatively comprehensive and quantitative analysis of *Plasmodium-*specific metabolites in supernatant from parasite infected culture system, and explores low molecular-weight biomarkers [6,13] associated with *Plasmodium.*

## Methods

### Parasite culture

In this study, asynchronized culture was employed. The purpose of using asynchronized culture was to capture small metabolite molecules that might be commonly released by all stages of parasites. The laboratory-adapted 3D7 strain of *P. falciparum* was used. The asynchronized blood stage parasites were cultured as described [14] in RPMI 1640 medium supplemented with 10% heat-inactivated O+ human serum, 1 ug/ml gentamicin, 36 uM hypoxanthine, 31 mM HEPES and 25 mM sodium bicarbonate. Four flasks of parasite culture with 3% haematocrit and 0.5% starting parasitaemia were prepared at the same time using red blood cells from different donors. At the same time, four flasks of culture without parasite inoculation but containing the same culture medium and haematocrit were also prepared. Culture materials, including supernatants and cell pellets, from all the infected and not infected flasks were collected at 12, 24, 36, and 48 hours without changing and adding culture medium. In total, 16 supernatant samples obtained from the infected flasks and the same numbers of supernatant samples from non-infected flasks were used for this study. All the samples used in this study were mycoplasma free. Parasite densities in the infected flasks were recorded as % count by blood smear reading. They increased with time, 0.83%±0.1%, 1.05%±0.06%, 1.45%±0.20% and 2.38%±0.30% (mean±SD) at 12, 24, 36 and 48 hours, respectively.

### The application of C18 liquid chromatography coupled with Fourier-Transform Mass Spectrometry (FTMS)

All the samples were run in duplicate. One hundred μl aliquots of supernatant samples were treated with acetonitrile (2:1, v/v), spiked with 2.5 μl internal standard mix, and centrifuged at 14,000 × g for 5 minutes at 4°C to remove protein as described previously [15]. Then LTQ-FTMS (Thermo, hybrid linear ion trap-Fourier Transform Ion Cyclotron Resonance mass spectrometry, MA, USA) coupled with C18 liquid chromatography was run on those collected samples. HRM offers an important advantage in analysis of highly complex metabolite mixtures, such as biological extracts, because detection of mass/charge (m/z) with 5 ppm or better mass resolution and mass accuracy substantially decreases the demand for physical separation prior to detection. Detection of m/z of ions from 85 to 850 with 50,000 resolution over 10 min LC runs with data extraction using apLCMS [16] provides a minimum of 3,000 reproducible features, many with sufficient mass accuracy to allow prediction of elemental composition. A m/z feature is defined by m/z, RT (retention time), and ion intensity (integrated ion intensity for the peak). The Kyoto Encyclopedia of Genes and Genomes (KEGG) database [17] was utilized to map the features distribution on both human and *Plasmodium* metabolic pathways. Examination of m/z of metabolites in the KEGG [18,19] human and *Plasmodium* metabolomics pathways shows that less than 10% of metabolites are redundant with others in terms of elemental composition [18,19]. Identified metabolites are annotated using Metlin Mass Spectrometry Database [20,21]. Direct examination by MS/MS of selected accurate mass m/z features of human plasma showed that for many m/z, the ion dissociation patterns matched those of authentic standards with identical elution times. For such metabolites, quantification relative to stable isotope internal standards has a coefficient of variation (5 to 10%) and sensitivity (low nanomolar to picomolar), which is similar to other methods and sufficient in allowing targeted analysis of selected chemicals within the context of an information-rich non-targeted profiling of all m/z detected within biological samples.

### Metabolic profiling with univariate and multivariate statistical analysis

Analyses were performed based on the results from both biological and technical replicates. Total features of culture supernatant were collected after processing mass spectral data with apLCMS. The features from duplicate LCMS analyses were averaged, log2 transformed and quantile normalized for subsequent statistical and bioinformatics analyses including univariate analysis, Manhattan plot, and false discovery rate (FDR) [22] to determine the significant metabolites between infected and non-infected cultures. Furthermore, the metabolic profiles were discriminated using Limma-hierarchical cluster analysis to separate two groups in association with metabolites. Limma is originally a package of Linear Models for Microarray to analyse the gene expression data arising from microarray or RNA-Seq technologies from Bioconductor. A core capability is the use of linear models to assess differential expression in the context of multifactor designed experiments. Limma provides the ability to make comparisons between many targets simultaneously including metabolites [23,24].

### Pathway analysis with KEGG

The database of Kyoto Encyclopedia of Genes and Genomes (KEGG) was utilized to map the features distribution on both human and plasmodium metabolic pathways. Detected m/z features matching known human and *Plasmodium* intermediary metabolites are shown in black dots in pathway maps; most human and known *Plasmodium* metabolic pathways are represented.

### Quantification of 3-methylindole and succinylacetone

3-methylindole (M51458) and succinylacetone (D1415) were purchased from Sigma-Aldrich (MO, USA). A standard curve was made by known concentrations (0.1-0.5nmole) of the reagent grade compounds in cell culture media. Areas from the samples were plotted against concentrations and a standard curve with an r^2^>0.99. 3-methylindole and succinylacetone in supernatants from parasite culture were treated using 100 μl in microcentrifuge tubes. These tubes were centrifuged at 14,000× g at 4 degrees Celsius for 5 minutes. 10 μl of the supernatant was injected in mass spectrometry and resulting areas were noted. Concentrations of the compounds were calculated using the standard curve.

## Results

### Metabolome-wide association study (MWAS)

**Figure 1:**
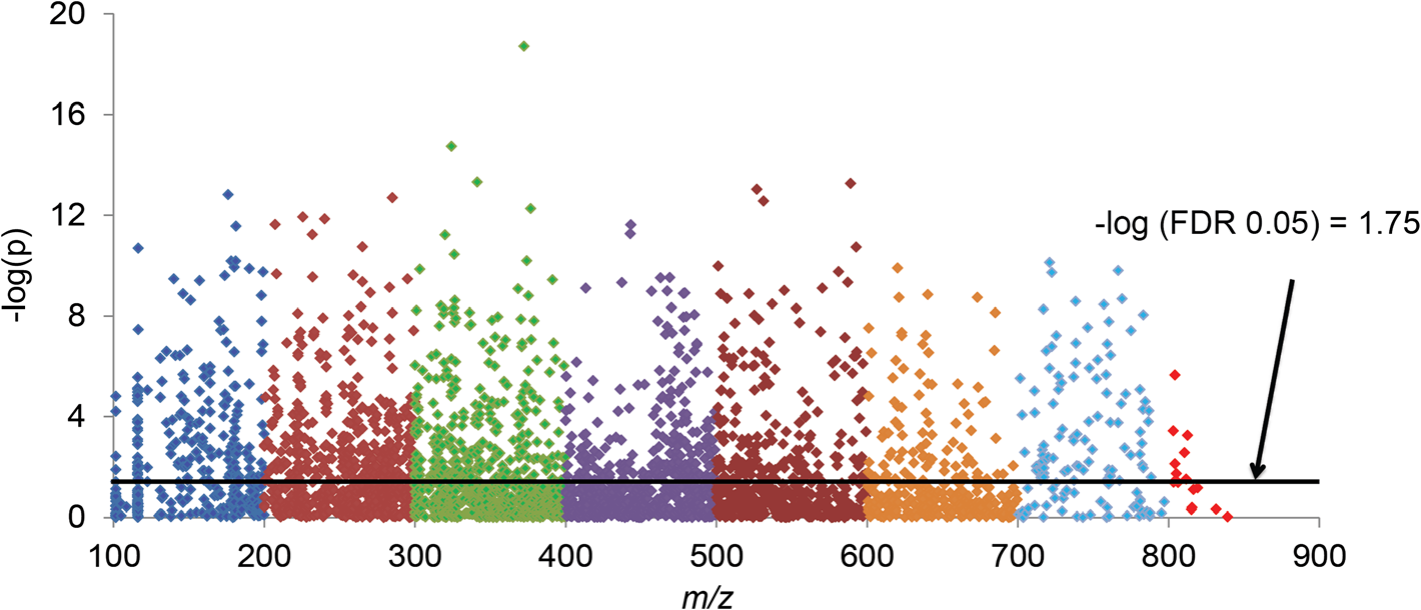
**Metabolome-wide association study (MWAS).** This is the snapshot of metabolites between supernatants from *Plasmodium,* non-infected and infected cultures at all time points using Manhattan plot on 3270 features. The features from duplicate run were averaged, log2 transformed, and quantile normalized to find out the significant features using false discovery rate (FDR). The dotted line represents FDR q=0.05. The metabolites over this line were the significant metabolites (n=1025) between two groups.

MWAS was used to identify changes in supernatants from non-infected and *Plasmodium*-infected cultures at all points (12, 24, 36, and 48 hrs). A Manhattan plot (Figure 1) combines a statistical test (e.g., p-value, ANOVA) with the magnitude of change and enables visual identification of statistically significant data-points (metabolites) that display large-magnitude changes. Multiple testing corrections like FDR adjusts p-values (q-values) derived from multiple statistical tests to correct for the occurrence of false positives. The Y axis represents the -log_10_ of the raw p-value comparing supernatants of culture system between non-infected and *Plasmodium*-infected red blood cells. The X axis indicated m/z ranging 85-850 m/z. The dotted line was shown as the FDR significant level, therefore, any m/z above this line were significantly different between two groups at FDR q=0.05. The total number of significant features was 1025 out of the 3270 detected.

### Mapping significant features through Kyoto Encyclopedia of Genes and Genomes (KEGG) human and plasmodium metabolic pathways

HRM platform provides precise metabolic phenotypes to determine the possible pathways altered by *P. falciparum*. The schematic representation in Additional files 1 and 2 shows that FDR significant 1025 features covering malaria parasite waste chemicals and human metabolites from supernatants were mapped through KEGG both human and *Plasmodium* metabolic pathways. Results show that these metabolites matched 439 metabolic compounds in both human and *Plasmodium* metabolic pathways. The remaining 586 of 1025 chemicals which were not matched, however, might be either waste products of the parasite or could be utilized by unidentified *Plasmodium* pathways.

### Two way hierarchical cluster analysis (HCA) on FDR significant features in supernatant between non-infected and infected cultures

**Figure 2:**
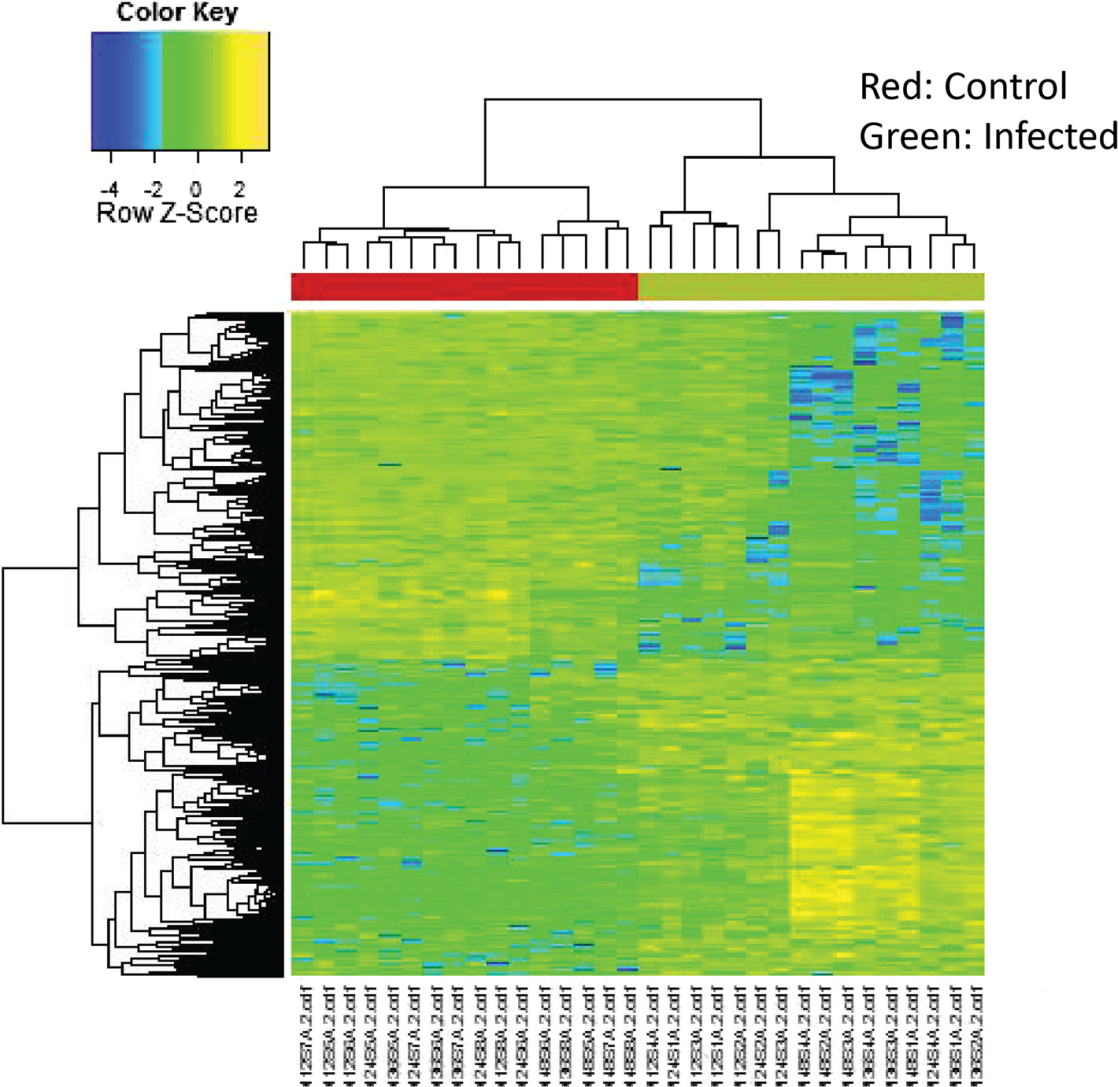
**Two way hierarchical cluster analysis (HCA) on FDR significant features of supernatants between non-infected and infected cultures.** HCA was performed using the 1025 metabolites at FDR q=0.05. The analysis utilized the samples from all time points to separate two groups in top bar (red clusters for control samples, green clusters for infected samples).

**Figure 3:**
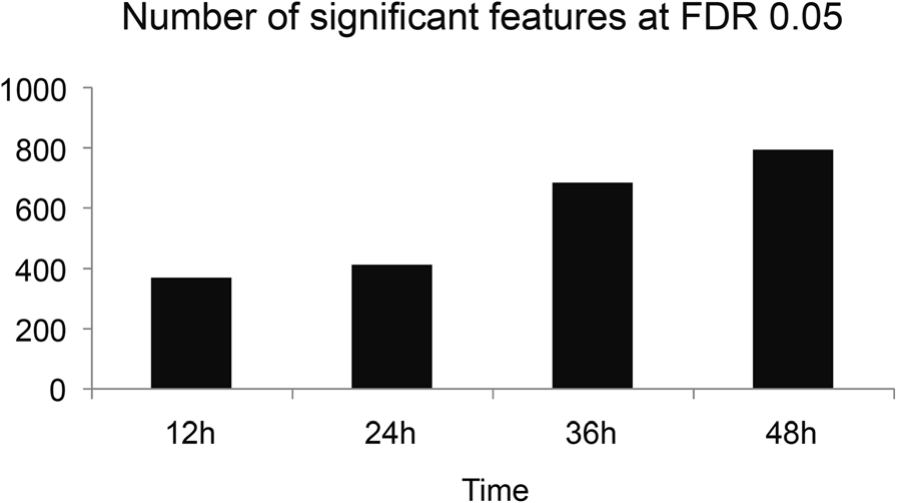
**The number of significant features using FDR q=0.05 at each time point during 48 hours’ culture.**

Two way HCA was performed on combined sample classification with metabolites clustering to identify which metabolites are the most important for sample grouping. In this study, HCA was performed using 1025 metabolites at FDR q=0.05, which were the key components to separate two groups using all four time points (Figure 2). HCA determines similarity measures using Euclidean distance and Pearson linear correlation. The top panel showed that two main clusters separating supernatant of non-infected from infected cultures. The sample name was listed at the bottom panel. The right panel included 1025 metabolites which contributed to discrimination of samples according to malaria infection. In addition, Figure 3 shows a broad increasing trend in significant features at FDR q=0.05 during 48 hours.

### Decrease in arginine and isoleucine in the agreement with others’ results

**Figure 4:**
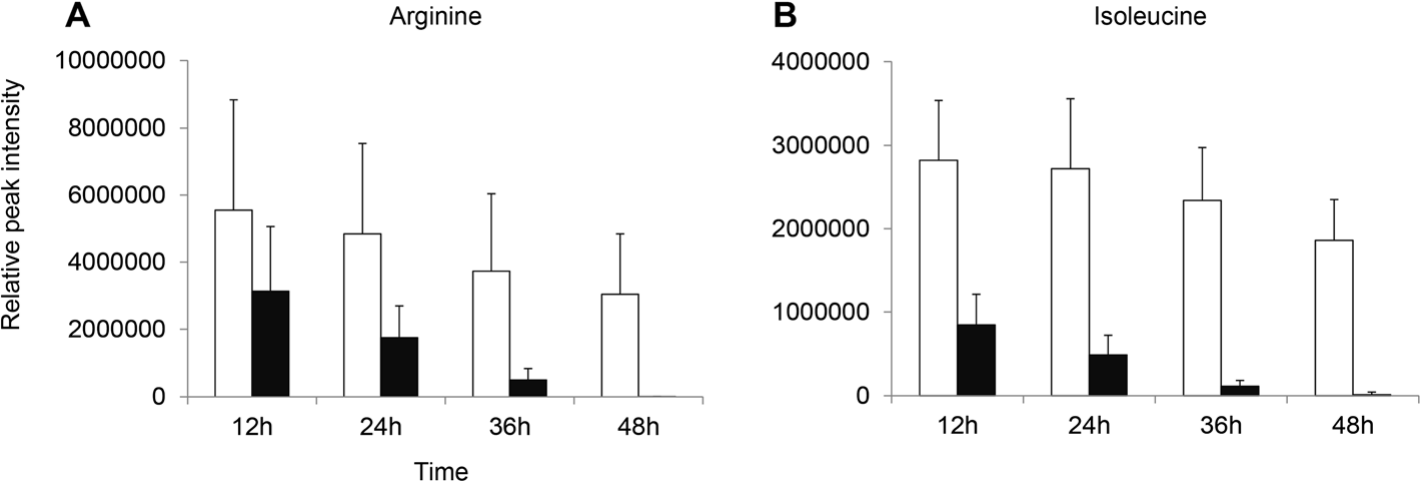
**Decrease of arginine and isoleucine concentration during 48 hours’ culture.** Figure 4 **A**: Arginine, and Figure 4 **B**: Isoleucine. White bars indicated supernatants from *Plasmodium* non-infected culture and black bar represented as supernatants from *Plasmodium* infected culture.

In order to validate methodology and analytical approach used for the identification of candidate biomarker molecules, the intensity of arginine and isoleucine known to be consumed and critical for malaria parasite growth were analysed. Amino acids are the building blocks of proteins. As parasites grow within host red blood cells, they utilize large quantities of amino acids. The malaria parasite derives most of its amino acid requirements from the proteolysed haemoglobin in the host blood cells [25]. In Figure 4A, arginine decreased to zero in a time dependent fashion during 48 hours. Most importantly, hemoglobin lacks one important amino acid, isoleucine, and the parasite, therefore, has to source this from culture system. In Figure 4B, isoleucine was reduced significantly in supernatants after the 48 hour incubation period.

### Identification of the potential biomarkers increased with culture time

**Figure 5:**
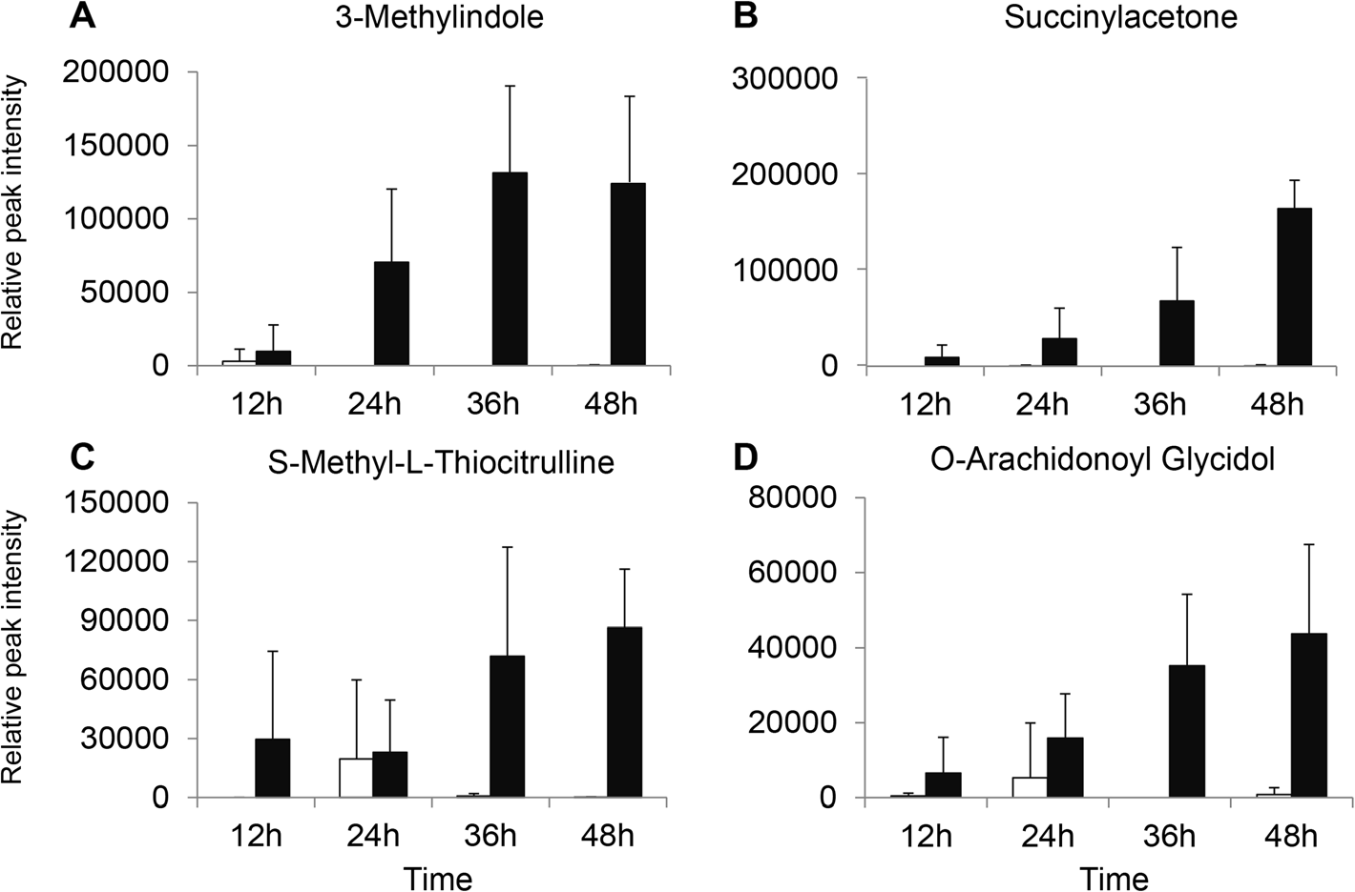
**Increase in concentrations of four molecules during 48 hours’ culture.** Figure 5 **A**: 3-methylindole, Figure 5 **B**: succinylacetone, Figure 5 **C**: S-methyl-L-thiocitrulline, and Figure 5 **D**: O-arachidonoyl glycidol. White bars indicated supernatants from *Plasmodium* non-infected culture and black bar represented as supernatants from *Plasmodium* infected culture.

Among the remaining 586 of 1025 significant metabolites (FDR q=0.05) determined by FDR, the ion intensities of several metabolites were increased with culture time in infected culture supernatants but not in non-infected culture supernatants, suggesting a positive association between the quantity of these molecules released and level of parasitaemia. These metabolites were: i) 3-methylindole (Figure 5A), ii) succinylacetone (Figure 5B), iii) S-methyl-L-thiocitrulline (Figure 5C), and iv) O-arachidonoyl glycidol (Figure 5D). The compound 3-methylindole has been shown to stimulate an odorant receptor to attract malaria mosquito vector [26] while succinylacetone has been identified as an inhibitor of haem biosynthesis [27,28]. S-methyl-L-thiocitrulline has been identified as a potent nitric oxide synthase (NOS) inhibitor to reduce nitric oxide production and endothelial dysfunction [29]. On the other hand, O-arachidonoyl glycidol was reported to be an inhibitor of fatty acid amide hydrolase [30,31]. In addition, a similar pattern was observed for phosphocholine (Additional file 3), the molecule that was reported in previous study using infected erythrocytes [11] and is involved in the phosphobase methylation for phosphatidylcholine production [32]. The observed increase in phosphocholine with culture time further validated our methodology and analytical approach for identifying the four molecules reported above.

### The quantification of 3-methylindole and succinylacetone

**Figure 6:**
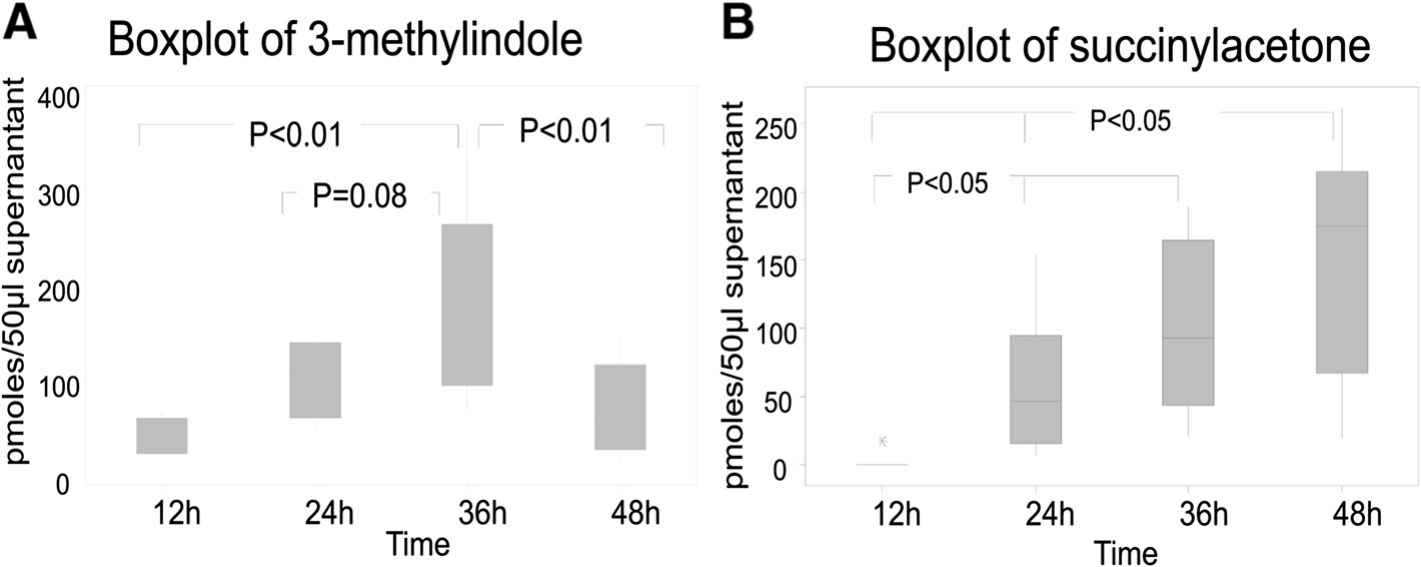
**The quantification of 3-methylindole and succinylacetone at each time point during 48 hours’ culture.** Figure 6 **A**: Boxplot of 3-methylindole, and Figure 6 **B**: Boxplot of succinylacetone.

The production of 3-methylindole and succinylacetone were measured at each time point during 48 hours in the 50 μl of supernatants from 3% haematocrit cultures. At 36 hours, the amount of 3-methylindole was highest and the concentration was 178±18.7 pmoles (Figure 6A). The generation of succinylacetone increased over the time. The amounts were 2±2 pmoles at 12 hours culture, with the highest reading at 157±30.5 pmoles at 48 hours culture (Figure 6B). Supporting information can be seen with the MS/MS data of these compounds found in Additional files 4, 5, 6 and 7.

## Discussion

The primary objective of this study was to explore *P. falciparum*-specific low molecular-weight metabolites that can be used as biomarkers for future development of non-invasive malaria diagnostic tools. Using the supernatant samples from *in vitro* erythrocytic stage asynchronized cultures, four molecules, 3-methylindole, succinylacetone, S-methyl-L-thiocitrulline and O-arachidonoyl glycidol, were identified as potential biomarkers.

Use of HRM is a uniquely good approach to identify putative biomarkers for a complex disease like malaria since *Plasmodium* parasites divert nutrients toward proliferating parasite cells while the host cells try to maintain homeostasis and deal with metabolic changes during the parasites’ intraerythrocytic life cycle [33,34]. This study demonstrated the strength of HRM in measuring *P. falciparum* specific waste products and toxins which were expected to increase in concentration during the infection. Therefore, this approach allows for the identification of potential biomarkers associated with *Plasmodium*-specific products. Previous studies also showed that the analytic capabilities of HRM could measure the relative levels of all metabolites simultaneously in *in vitro* and *in vivo* malaria infection systems [12]. Furthermore, using the outcomes of HRM, KEGG mapping was performed in this study. Significant features (n=1025) were identified in both human and *Plasmodium* metabolic pathways to distinguish which metabolic compounds are being utilized by both. Surprisingly, 439 metabolites were found to be used in both human and plasmodium metabolic pathways. However, despite the similarities in this large number of metabolites, the pathways in which these metabolites are mapped are likely to be different due to the absence of certain metabolic pathways in plasmodium compared to human metabolic pathways. Meanwhile, of the 586 unmatched features, four (4) were found to be potential biomarkers from the parasite during the erythrocytic stage culture system. This was based on the fact that the ion intensities of the four molecules were increased with culture time, suggesting a positive association between relative quantity of these molecules and level of parasitaemia. In addition, 3-methylindole and succinylacetone molecules in the supernatant samples from 3% haematocrit 48 hour time-course cultures were further quantified. The concentration of succinylacetone peaked at 48 hours compared to 36 hours for 3-methylindole. As 3-methylindole is volatile molecule, it is possible that 3-methylindole was evaporated due to extended culture to 48 hours. Importantly, 3-methylindole has been shown to stimulate an odorant receptor to attract malaria mosquito vector [26], potentially playing a role in enhancing the probability of transmission for the parasite. Practically, 3-methylindole could be used in traps for mosquito research purposes. Although 3-methylindole is found in large intestine of humans via ingestion of tryptophan, this molecule is well-known as a highly selective pulmonary toxicant for ruminants [35]. A study showed that metabolism and bioactivation of 3-methylindole in human is mediated by human liver microsomes [36]. In spite of the fact that this molecule is found in human gastrointestinal system, it is probable that the elevation of this molecule due to malaria infection in human could still be used as a potential biomarker when urine or sweat samples are tested.

Another potential biomarker identified was succinylacetone which is known to inhibit haem biosynthesis (delta-aminolevulinate dehydrolase inhibitor) [37]. Succinylacetone either makes the haem synthesizing system nonfunctional or decreases its functionality [37]. Although this molecule has been shown to be a biomarker for Tyrosinaemia Type 1 (Tyr I), the genetic disorder is rare and worldwide prevalence is very low. Therefore, utilizing this compound as a potential biomarker in malaria-endemic areas could account for the malaria infection rather than the Tyr I. The production range of succinylacetone suggests it may be useful as a basis for developing a biosensor for non-invasive diagnosis of malaria. Two other potential metabolites were also identified in this study: S-methyl-L-thiocitrulline is a potent NOS inhibitor to reduce nitric oxide production and endothelial dysfunction [38] while O-arachidonoyl glycidol was reported to be an inhibitor of fatty acid amide hydrolase [30]. The increase in ion intensities during culture period for these two molecules indicated a potential as biomarkers for malaria diagnosis. Currently, a metabolomics study using urine, saliva, sweat and blood samples collected from *P. falciparum* infected people from Africa is underway. The ongoing study will provide information on concentration and stability of the molecules in malaria infected people and their association with parasite densities.

An apparent disappearance of two amino acids, arginine and isoleucine, was observed in the 48 hr culture system. Several important host-parasite interaction studies elucidated a mechanism that isoleucine was taken by *Plasmodium* sp. to develop blood stage parasites [25,39]. Malaria parasite utilizes amino acids largely through the degradation of host erythrocyte haemoglobin [40]. Isoleucine is the only amino acid not present in human mature haemoglobin, resulting in utilization of this amino acid from other sources by parasites. The untargeted HRM confirmed previous finding regarding the depletion of isoleucine in culture supernatant in time dependent manner, supporting the reliability of experimental system and analytical approach in identifying the above four parasite-specific waste metabolites and toxins which were released into culture supernatant and were shown increase in concentration during 48 hr culture period.

In summary, HRM coupled with network and pathway analysis using the significant metabolites from culture supernatants of infected erythrocytes and incorporating the broader human and malaria parasite metabolomic knowledge identified four potential parasite-specific biomarkers. The findings from the current study may provide improved opportunities for innovative prevention and management programmes such as development of new malaria diagnostic tools.

## Additional files

Additional file 1:
**Mapping significant features to Kyoto Encyclopedia of Genes andGenomes (KEGG) human metabolic pathways.** This schematic representation shows the mapping of 439 matched features covering human metabolites. Additional file 2:
**Mapping significant features to Kyoto Encyclopedia of Genes and Genomes (KEGG)** ***Plasmodium*** **metabolic pathways.** This schematic representation shows the mapping of 439 matched features covering malaria parasite metabolites. Additional file 3: Increase in phosphocholine concentration during 48 hours culture. White bars indicated supernatants from *Plasmodium* non-infected culture and black bar represented as supernatants from *Plasmodium* infected culture. Additional file 4:
**Spectrum of 3-methylindole itself.** A) Total ion chromatography, B) MS, and C) MS/MS. Additional file 5:
**Spectrum of 3-methylindole after its addition to cell supernatant.** A) Total ion chromatography, B) MS, and C) MS/MS. Additional file 6:
**Spectrum of 3-methylindole in cell supernatant.** A) Total ion chromatography, B) MS, and C) MS/MS. Additional file 7:
**Spectrum of succinylacetone.** A) Total ion chromatography, B) MS on itself, C) MS/MS on itself, D) MS/MS after its addition to cell supernatant, and E) MS/MS on cell supernatant without chemical addition.
