# Supplementary figures and images for: High-resolution metabolomics to discover potential parasite-specific biomarkers in a Plasmodium falciparum erythrocytic stage culture system
Source: Malar J. 2015 Mar 24;14:122. doi: 10.1186/s12936-015-0651-1 (PMC4377044; doi:10.1186/s12936-015-0651-1)

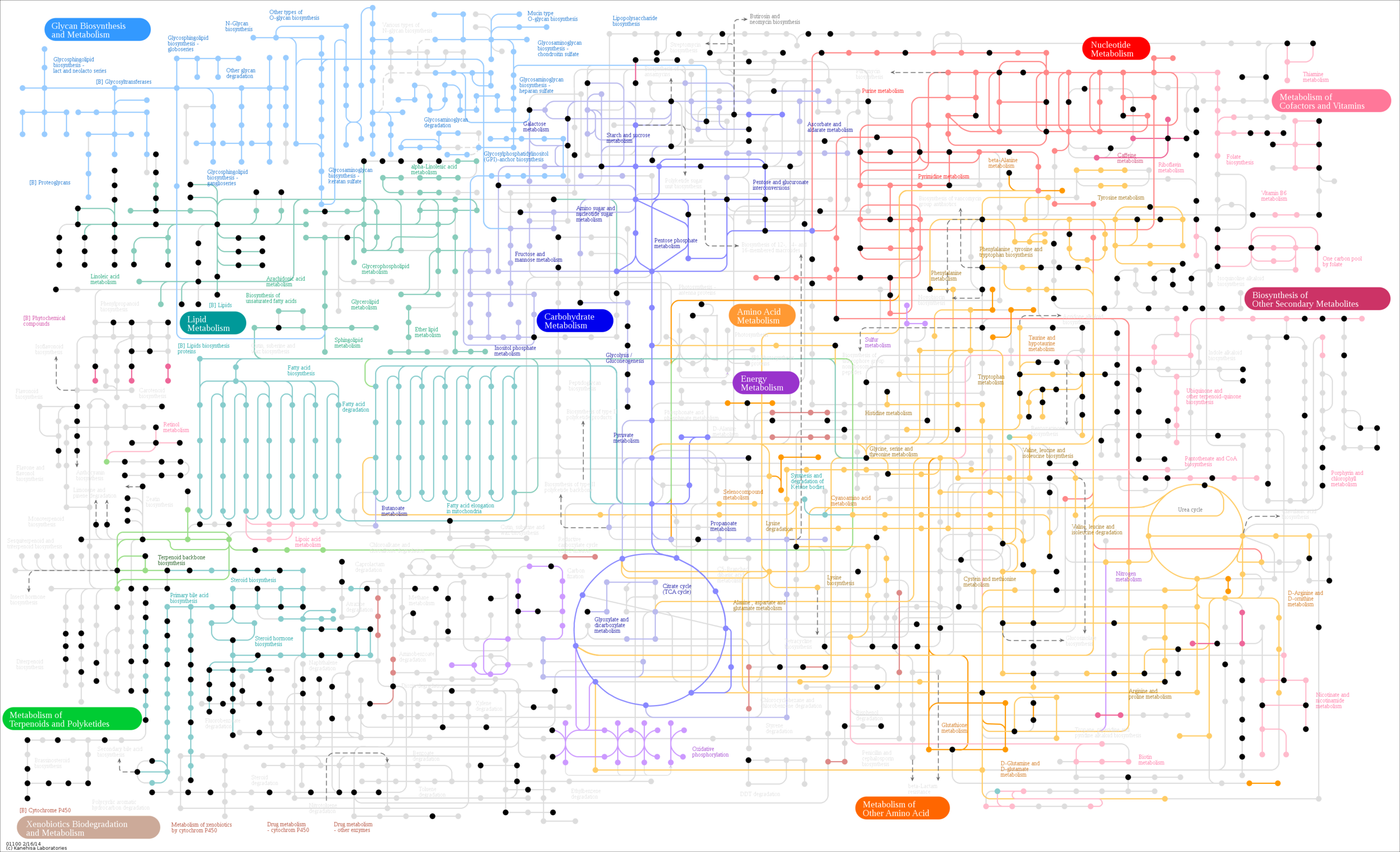

Supplement: Additional file 1: — Mapping significant features to Kyoto Encyclopedia of Genes andGenomes (KEGG) human metabolic pathways. This schematic representation shows the mapping of 439 matched features covering human metabolites. [file 12936_2015_651_MOESM1_ESM.png]

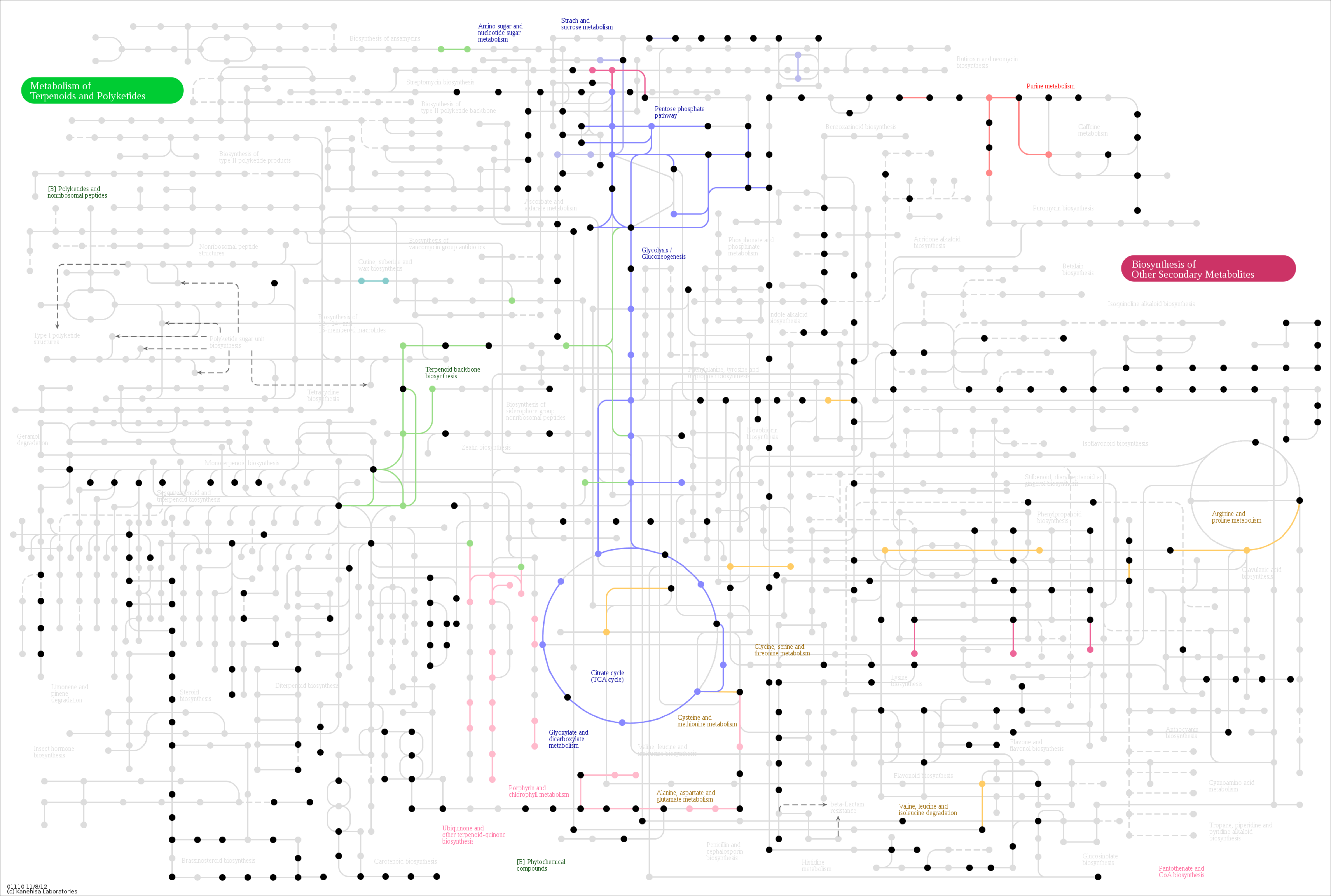

Supplement: Additional file 2: — Mapping significant features to Kyoto Encyclopedia of Genes and Genomes (KEGG) Plasmodium metabolic pathways. This schematic representation shows the mapping of 439 matched features covering malaria parasite metabolites. [file 12936_2015_651_MOESM2_ESM.png]

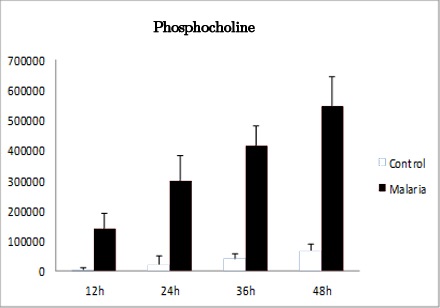

Supplement: Additional file 3: — Increase in phosphocholine concentration during 48 hours culture. White bars indicated supernatants from Plasmodium non-infected culture and black bar represented as supernatants from Plasmodium infected culture. [file 12936_2015_651_MOESM3_ESM.jpeg]

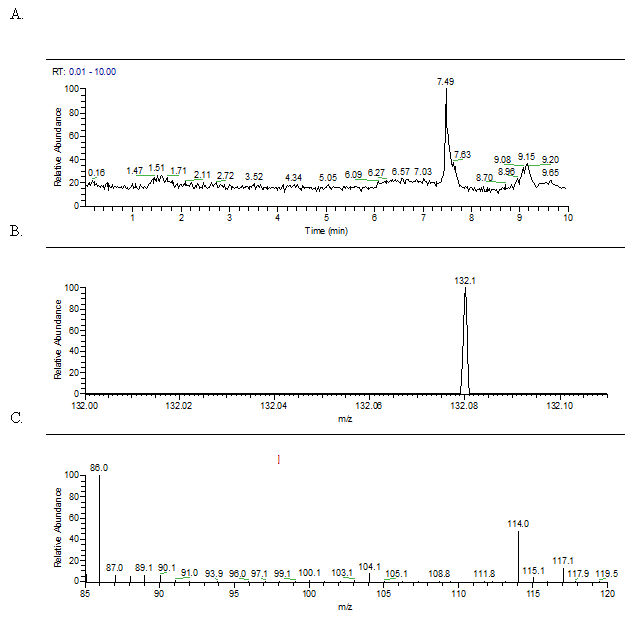

Supplement: Additional file 4: — Spectrum of 3-methylindole itself. A) Total ion chromatography, B) MS, and C) MS/MS. [file 12936_2015_651_MOESM4_ESM.png]

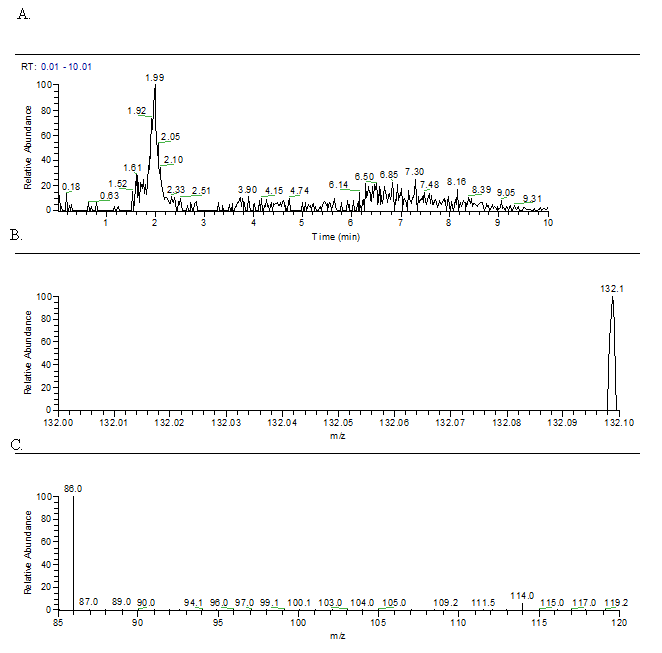

Supplement: Additional file 5: — Spectrum of 3-methylindole after its addition to cell supernatant. A) Total ion chromatography, B) MS, and C) MS/MS. [file 12936_2015_651_MOESM5_ESM.png]

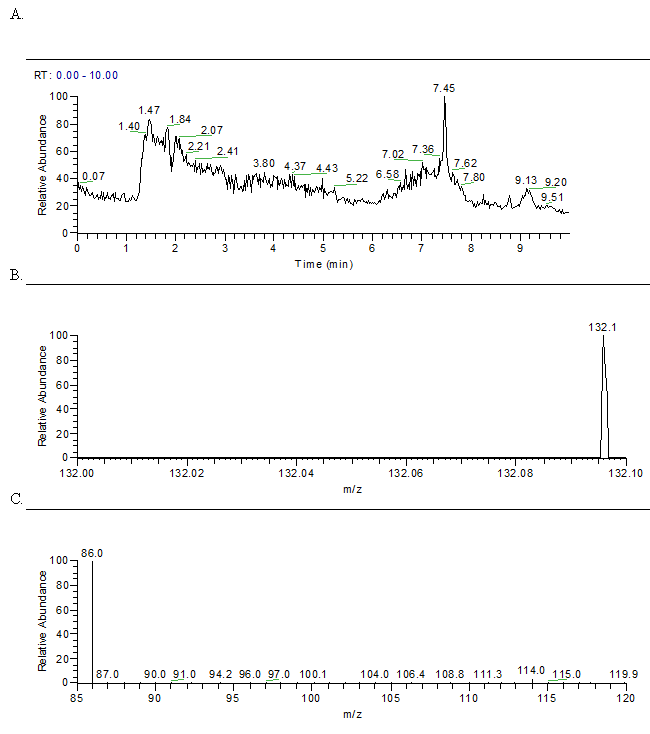

Supplement: Additional file 6: — Spectrum of 3-methylindole in cell supernatant. A) Total ion chromatography, B) MS, and C) MS/MS. [file 12936_2015_651_MOESM6_ESM.png]

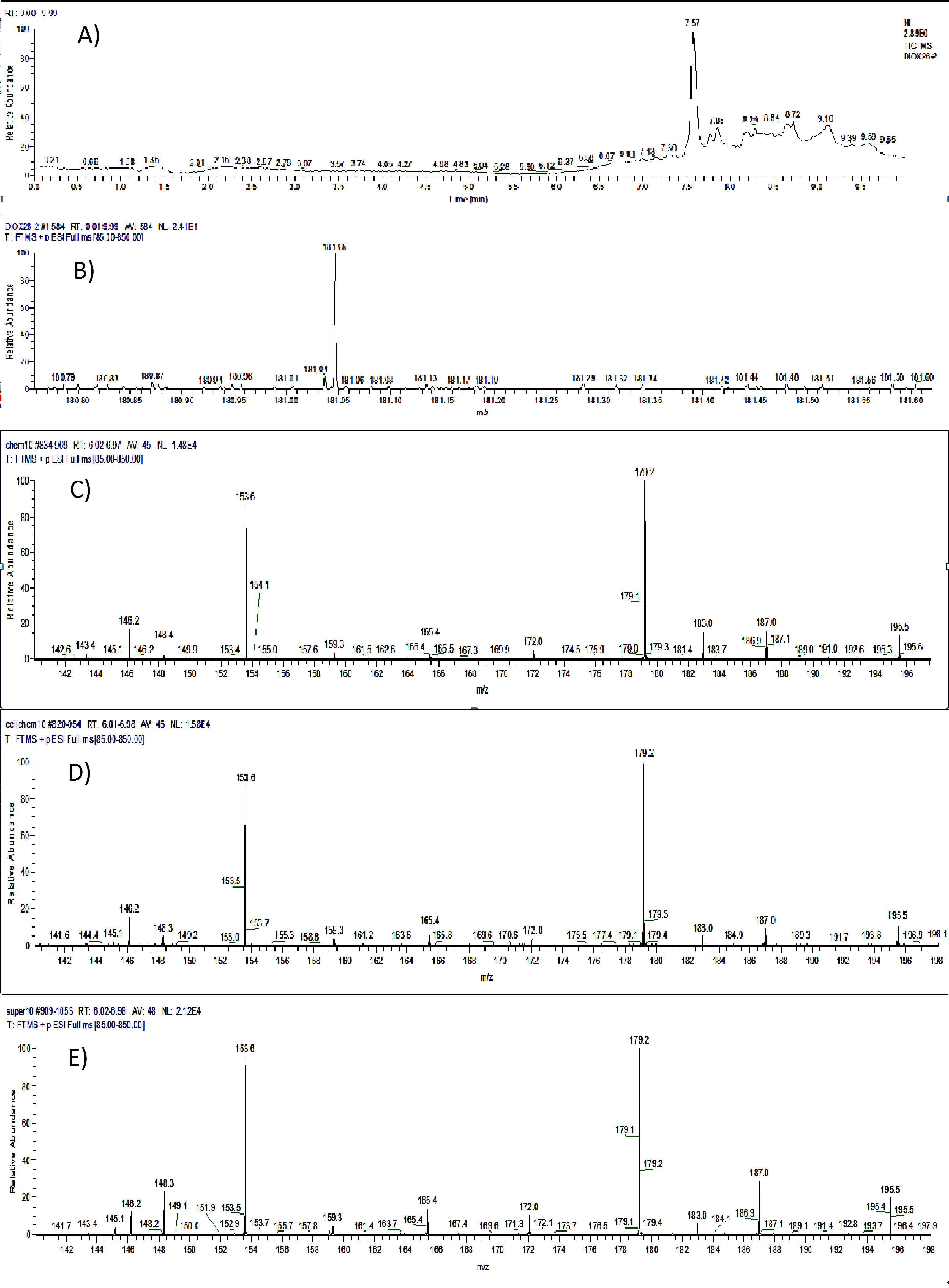

Supplement: Additional file 7: — Spectrum of succinylacetone. A) Total ion chromatography, B) MS on itself, C) MS/MS on itself, D) MS/MS after its addition to cell supernatant, and E) MS/MS on cell supernatant without chemical addition. [file 12936_2015_651_MOESM7_ESM.jpeg]
